# Impaired Verbal Memory Recall in Patients With Axonal Degeneration and Serum Glycine-Receptor Autoantibodies—Case Series

**DOI:** 10.3389/fpsyt.2021.778684

**Published:** 2022-01-28

**Authors:** Niels Hansen, Claudia Bartels, Winfried Stöcker, Jens Wiltfang, Dirk Fitzner

**Affiliations:** ^1^Department of Psychiatry and Psychotherapy, University Medical Center Göttingen, Göttingen, Germany; ^2^Euroimmun Reference Laboratory, Lübeck, Germany; ^3^German Center for Neurodegenerative Diseases (DZNE), Göttingen, Germany; ^4^Neurosciences and Signaling Group, Department of Medical Sciences, Institute of Biomedicine (iBiMED), University of Aveiro, Aveiro, Portugal; ^5^Department of Neurology, University Medical Center of Göttingen, Göttingen, Germany

**Keywords:** cognitive impairment, glycine receptor antibody, neurodegeneration, tau protein, memory recall

## Abstract

**Background:**

Glycine receptor antibody-associated neuropsychiatric disease is currently known to be dominated by the phenotypes stiff-person syndrome and progressive encephalomyelitis entailing rigidity and myoclonus. In our case series we aim to depict the less-often reported feature of cognitive impairment associated with glycine receptor antibodies.

**Methods:**

We investigated five patients with cognitive impairment varying from mild cognitive impairment to dementia associated with serum glycine receptor antibodies. Mild and major neurocognitive disorders were diagnosed according to the DSM-5 (fifth edition of the Diagnostic and Statistical Manual of Mental Disorders). Neuropsychology via Consortium to Establish a Registry for Alzheimer's Disease (CERAD) testing results, psychopathology data via the Manual for the Assessment and Documentation of Psychopathology in Psychiatry (AMDP), cerebrospinal fluid analysis and magnetic resonance imaging data were retrospectively analyzed from patient files.

**Results:**

We identified five patients with cognitive impairment as the main neuropsychiatric feature associated with serum glycine receptor antibodies. One patient also presented akinetic rigidity syndrome. The psychopathology comprised disorders of attention and memory, orientation, formal thought, and affect. In addition to suffering deficits in verbal memory function, figural recall, phonematic fluency, and globally deteriorated cognitive function, these patients presented seriously impaired memory recall in particular. Tau protein and phosphorylated tau protein 181 were elevated in 75% of patients.

**Conclusions:**

Our results suggest that axonal neurodegeneration and especially impaired verbal memory recall in addition to deficits in verbal and figural memory characterize patients with progressive cognitive impairment associated with glycine receptor antibodies. This unresolved issue should be clarified by researchers to discover whether axonal degeneration is merely an age-related phenomenon or one related to glycine-receptor autoantibodies in old age. Cognitive impairment as a neuropsychiatric syndrome of glycine-receptor antibody disease is a potential, conceivable, but so far unproven additional feature requiring deeper large-scale investigations and consideration during differential diagnosis in memory clinics.

## Introduction

Glycine-receptor autoantibodies are often detected in patients with stiff-person syndrome (1, 2)–a disease causing muscle spasms and rigidity and sometimes task-specific phobia (3). Another disease entity called progressive encephalomyelitis involving rigidity and myoclonus (PERM) is also known to be often associated with glycine receptor antibodies (4). Stiff-person syndrome associated with glycine-receptor autoantibodies varied widely by age (12–62) (1), but it mainly affects people in middle age (1). Glycine receptors play a major role in inhibitory synaptic transmission in the central nervous system (5, 6). There is evidence that autoantibodies against the glycine receptor result in stiffness, myoclonus, and rigidity (7) due to failing inhibitory synaptic transmission (5). Less is known about the clinical symptoms of cognitive dysfunction associated with glycine-receptor antibodies. Recent reports suggest that a minority of patients with glycine-receptor antibodies are cognitively impaired (8). Our case series aimed to describe a homogeneous group of older, not middle-aged patients with cognitive decline in whose serum we had also detected glycine-receptor autoantibodies that might be phenotypically different from patients typically affected by stiff-person syndrome in association with glycine-receptor antibodies. Furthermore, we aim to examine whether axonal neurodegeneration can be detected in patients with glycine receptor-related cognitive impairment or disease. Our case series demonstrates a homogeneous pattern of cognitive impairment entailing major deficits in verbal memory recall as well as tau protein-based neurodegeneration in patients with glycine-receptor antibodies.

## Methods and Patients

We investigated five patients from the Department of Psychiatry and Psychotherapy and Neurology University Medical Center Göttingen presenting serum glycine-receptor antibodies and cognitive impairment extending from mild cognitive impairment (MCI) to dementia. Dementia (major neurocognitive disorder) was diagnosed according to the definition of the fifth edition of the Diagnostic and Statistical Manual of Mental Disorders (DSM-5) (9) involving higher cortical dysfunction in addition to disability in daily living. To categorize the severity of cognitive impairment, we applied the DSM-5 classification distinguishing between mild and major neurocognitive disorder. A mild neurocognitive disorder corresponds to the term MCI and was diagnosed if the patient exhibited a mild neurocognitive disorder and had no disability or problems with daily activities. We applied the Consortium to Establish a Registry for Alzheimer's Disease Plus (CERAD) for neuropsychological assessment. Z-score calculations relied on normative data and the translated German version of the CERAD. We used a CERAD software (CERAD-Plus) that calculated z-score results without additional analysis from our staff. Patients' CSF probes were examined in the Neurochemistry Laboratory of the Department of Neurology, University Medical Center Göttingen. The following in-house, unpublished normative values established in the Neurochemistry Laboratory of the Department of Neurology, University Medical Center Göttingen were used to determine if the biomarker levels were within the non-pathological range. Non-pathological values were declared when we detected these constellations: (a) tau protein: <450 pg/ml, (b) phosphorylated tau protein 181 (ptau181): <61 pg/ml, (c) ß-amyloid 42 (Aß42): >450 pg/ml, and (d) ratio Aß42/Aß40 × 10: >0.5. The levels of CSF total tau and phosphorylated tau protein 181 (ptau181) were manually quantified using the commercial enzyme-linked immunosorbent assay (ELISA) from Fujirebio [INNOTEST hTAU-Ag; INNOTEST PHOSPHO TAU (181P)]. CSF Biomarker Aß42 was manually measured utilizing the commercially available INNOTEST® β-AMYLOID (1–42) ELISA kit (Fujirebio). CSF Aß40 was manually measured through commercially available ELISA from IBL [AMYLOID BETA (1–40)]. Antibodies against the alpha 1 glycine receptor were determined taking an immunocytochemical approach by transfecting cells with the glycine receptor alpha1 subunit and later fixation of those cells on arrays used for analysis in the Euroimmun Laboratory, Lübeck. We examined the following antibodies in all patients in serum and the CSF: anti-α-amino-3-hydroxy-5-methyl-4-isoxazolepropionic acid receptors 1/2 (anti-AMPAR1/2), -Amphiphysin, -Aquaporin 4, -contactin associated protein 2 (CASPR2), -CV2, -dipeptidyl-peptidase-like 6 protein (DPPX), -Flotillin 1/2, -gamma aminobutyric acid B1/2 receptor (GABAB1/2R), -glutamic acid decarboxylase (GAD65), -HuD, -LGI1, -Ma1/ Ma2, -N-methyl-D-aspartate receptor (NMDAR), -Ri, -SOX1, -TR, -Yo, and -Zic4 antibodies. Psychopathology was assessed by the Manual for the Assessment and Documentation of Psychopathology in Psychiatry (AMDP) (10). 1.5 T MRIs were done in the Department of Neuroradiology, University Medical Center Göttingen or off-site at neuroradiologic centers and visually evaluated by neuroradiologists. No specific algorithm was used to quantify brain atrophy. The patient's age was considered when assessing brain atrophy. Clinical features were extracted retrospectively from patient files. Autoimmune indicators were adapted from recent guidelines on autoantibody-associated psychiatric syndromes (11). This study was conducted in agreement with the latest version of the Declaration of Helsinki and was approved by the Ethics Committee of the University Medical Center Göttingen.

## Results

### Clinical Characterization of Patients

Our patients had an average age of 72 ± 4 years (3 females) (Table 1) and an age at disease onset of 64 ± 5 years. All had serum autoantibodies against glycine receptors. Three patients revealed an MCI, and two had dementia. The patients' neuropsychiatric syndromes ranged from cognitive dysfunction to idiopathic Parkinson's syndrome. Additional neuropsychiatric comorbidities were present ranging from carpal tunnel syndrome to polyneuropathy. Other comorbidities are depicted in Table 2. According to their sum scores in decreasing order, our patients' psychopathologies revealed disorders of attention and memory, affect, formal thought, and orientation (Table 1). Not apparent were disorders of consciousness, worries and compulsions, delusions, disorders of perception, ego disturbances, circadian disturbances, social withdrawal, excessive social contact, aggressiveness, suicidal behavior, self-harm, lack of feeling ill, lack of insight into illness, and uncooperativeness. Strong indicators of autoimmunity were present in 1 patient (20%) with abnormal movements, and in another patient (20%) with paresthesia (Table 1). These strong indicators of autoimmunity were not apparent: aphasia, mutism, dysarthria, autonomic disturbances, central hypoventilation, decreased level of consciousness, epileptic seizures, faciobrachial dystonic seizures, focal neurological deficit, hyponatremia, infectious prodrome, new-onset headache, adverse response to antipsychotic or antidepressant drugs, optic hallucinations, other autoimmune disorder, presence of a tumor or a neuroleptic malignant syndrome. No patient revealed a weak indicator of autoimmunity such as confusion, a dynamic course, early resistance to therapy, or fluctuating psychopathology. Brain MRIs revealed generalized brain atrophy in 2 of 5 (40%) and focal brain atrophy in 1 of 5 (20%) patients. The focal atrophy in one patient affected mainly the hippocampal formation together with mild general brain atrophy. Vascular lesions were detected more often in 3 of 5 (60%) patients, but they were clinically not symptomatic, as the affected patients had suffered no ischemic attacks or enduring focal neurological deficits.

**Table 1:** Clinical characterization of patients.

**Parameter**	
**Demographic parameter**	
Sex (female)	3/5
Age y	72 ± 3.7
Onset y	64.3 ± 4.5
Early-onset	2/5
**Psychopathology**	
Disturbances of orientation	1/5
Disturbances of attention and memory	5/5
Formal thought disorder	4/5
Disturbances of affect	4/5
Disorders of drive and psychomotor activity	1/5
**Strong indicators for autoimmunity**	
Movement disorder	1/5
Paresthesia	1/5
**CSF**	
Cell count (<5 μg/L)	0 ± 0
Albumin mg/L	739.75 ± 348
IgG mg/L	77.65 ± 44.6
IgA mg/L	10.8 ± 7.67
IgM mg/L	1.33 ± 0.89
**Brain MRI**	
Generalized atrophy	2/5 (40%)
Focal atrophy	1/5 (20%)
Vascular lesions	3/5 (60%)

*AD, Alzheimer's disease dementia; CERAD, The consortium to establish a registry for Alzheimer's disease; CSF, cerebrospinal fluid; GDS, Geriatric depression scale; IgA, immunoglobulin A; IgG, immunoglobulinG; IgM, immunoglobulin M; MMSE, mini mental status examination; MRI, magnetic resonance imaging; NABD, neural autoantibody-associated dementia; P Tau Protein 181, phosphorylated tau protein 181; y, years. The values are depicted as mean ± standard deviation*.

**Table 2:** Clinical characterization of neuropsychiatric syndromes and comorbidities in patients with glycine receptor antibodies.

**Patient**	**Neuropsychiatric syndrome**	**Comorbidity**
1	Amnestic MCI	Arthrosis
2	Amnestic MCI	Aterial hypertension, prior Non-Hodgkin lymphoma
3	Amnestic MCI, depressive disorder	Polyathrosis, hypercholesterinemia, arterial hypertension, prior TIA
4	Dementia	Hypertension, diabetes mellitus type II
5	Dementia, IPS	Carpal tunnel syndrome, polyneuropathy

*MCI, mild cognitive impairment; IPS, idiopathic Parkinson's syndrome; TIA, transient ischemic attack*.

### Cognitive Function in Patients

**Figure 1:**
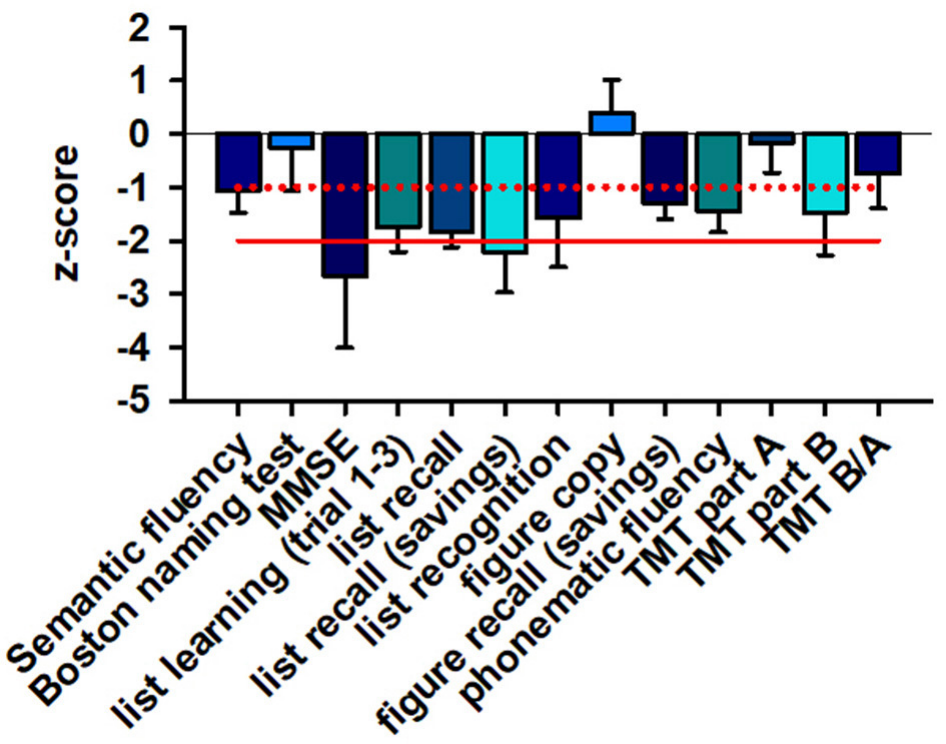
Neuropsychological data. MMSE sum scores and verbal memory recall are severely affected in patients, whereas verbal and figural memory, phonematic fluency, cognitive flexibility (TMTB) are only mildly affected. The z-score of different cognitive subdomains is depicted. The red line indicates the −2 z-score value whereas the dotted red line shows the −1 z-score value. A ≤ −2 z-score value is regarded as major cognitive dysfunction whereas a ≤ −1 value is as considered minor cognitive dysfunction. MMSE, mini mental status examination; TMT, trial making test.

CERAD testing revealed relevant deficits in four patients' word list learning and word list recall as well as word saving (Figure 1). Their saving recall (verbal memory recall) was more impaired than other cognitive subdomains, apart from the general cognitive assessment via the MMSE in four patients (Figure 1). Less pronounced, but also affected were cognitive flexibility (evident through the Trial Making Test (TMT) part B) and figural memory (measured by impaired figure recall) as well as phonematic fluency. The patients' visuoconstructive capacity was only mild affected.

### Neurodegeneration Markers in Patients

**Figure 2:**
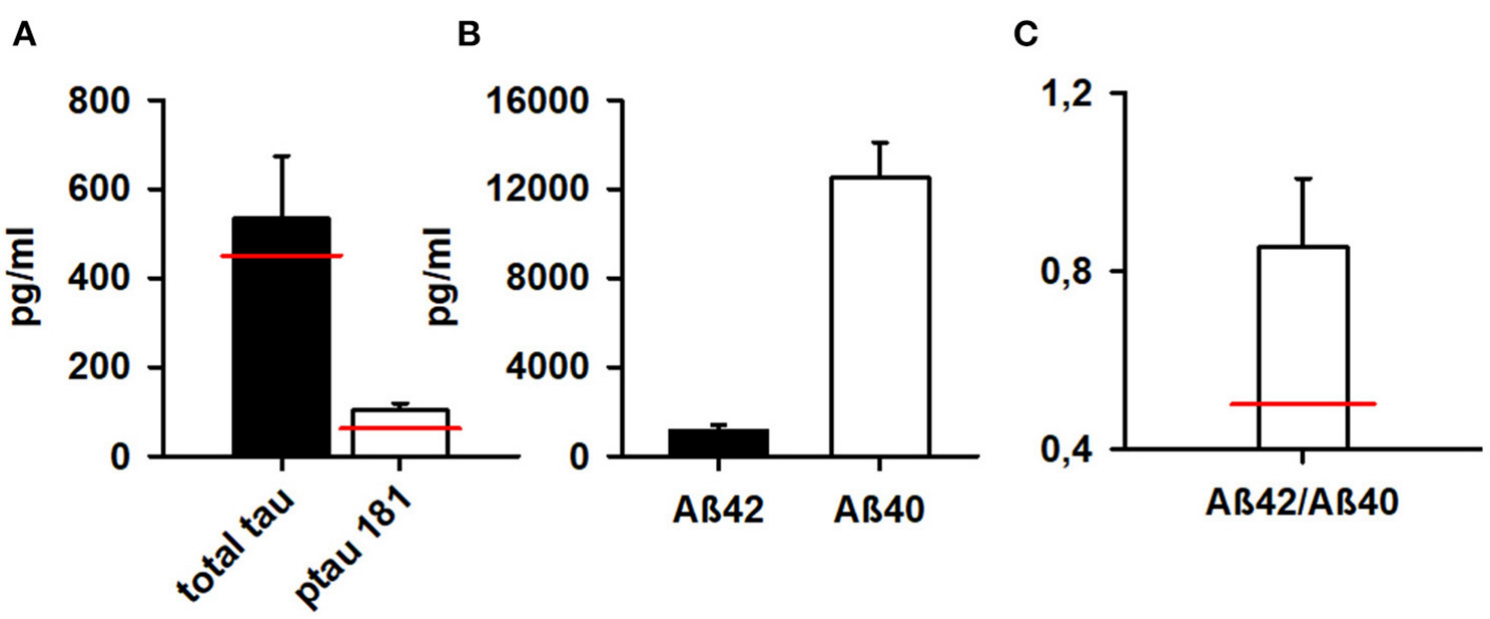
Markers of neurodegeneration in patients. Phosphorylated tau protein 181 and total tau protein are above normal levels **(A)** whereas Aß42 and **(B)** Aß42/Aß40 ratio **(C)** are normal according to our normative reference values from the Neurochemistry Laboratory (see methods). Aß42, ß-amyloid 42; Aß40, ß-amyloid 40; Aß42/Aß40, ß-amyloid 42/40. The red bar indicate normal levels of neurodegenerative markers.

Both total tau protein and phosphorylated tau protein 181 were elevated in 3 of 4 (75%) patients in our patient cohort (see Table 1; Figure 2). Aß42 and the Aß42/40 ratio were not below normal levels (Figure 2).

## Discussion

Our case series from a memory clinic confirms that glycine-receptor antibodies in patients with cognitive impairment ranging from MCI to dementia seems to be a valuable phenotype not yet reported on extensively as differing from the PERM phenotype usually described to characterize glycine-antibody disease. Furthermore, our patient group's age differs from what is usually noted in patients with stiff-person syndrome, as they tend to be much younger, although the ages when affected can vary widely, as in a recent case series (1). Thus, the glycine-receptor antibody in older age might be accompanied by various phenotypic spectrums associated with cognitive impairment that differ from classical stiff-person syndrome or the PERM phenotype. The glycine receptor antibodies in our case series target the glycine receptor alpha1 unit–the main known target of glycine receptor antibodies (12). The disease entity PERM is often predominant in patients with glycine receptor antibodies, as shown in two large patient cohorts (8, 13). Limbic encephalitis, encephalitis or encephalopathy are less often reported in conjunction with glycine-receptor antibodies in these studies. One of our patients suffers from akinetic rigidity syndrome, in line with a report of a Parkinson's syndrome involving frontal lobe atrophy and glycine-receptor antibodies (14). However, recent research showed novel features in a subgroup of glycine-receptor antibody-positive patients describing various visual dysfunction phenomena (15). The broad variability of glycine-receptor antibody-associated diseases is not surprising, and reveals the need to further examine and specify glycine-receptor-associated phenotypes. There are few reports of clinical phenotypes, but certain common features of glycine-receptor antibody disease have been described, such as PERM and akinetic rigidity syndrome. We detected more generalized brain atrophy in most of our patients; only two presented focal atrophy. Concurring with these signs of neurodegeneration in brain MRI, our CSF results deliver support, as we detected axonal degeneration in 75% via elevated tau and tau 181 protein. Axonal neurodegeneration may coincide with autoantibody-associated cognitive decline (16). Verbal memory involvement, with particular focus on memory recall in our patient with glycine-receptor antibodies is highly likely, as glycine receptors are expressed in the hippocampus (17). Animal neuronal cultures and patch clamp investigations (6) have shown that glycine-receptor antibodies against the extracellular domain and N-terminus (the main autoantibody epitope) impair glycine-receptor function. The chloride ion channel is blocked by glycine-receptor antibodies, and glycine's potency is reduced as higher concentrations of glycine are required when glycine-receptor antibodies are blocked, as the aforementioned working group demonstrated. Inhibitory transmission in the hippocampus is disturbed by glycine-receptor antibodies–similar to observed alterations in inhibitory synaptic transmission in the spinal cord and evident in a study using patch clamp technique in spinal cord neurons (5). Furthermore, the presence of glycine-receptor autoantibodies in zebrafish serum is known to reduce the glycine-receptor cluster in zebrafish larvae (6) and to induce the internalization of glycine receptors in neurons (18). It is thus conceivable but still unproven that if the blood-brain barrier is leaky and glycine-receptor antibodies can enter the brain including hippocampal tissue, the distribution of glycine receptors (caused by potentially-internalized glycine receptors from the neuronal cell surface) might be altered, which would then impair hippocampal synaptic information processing for longer time intervals. Two facts should be discussed regarding glycine-receptor antibody-associated cognitive impairment: first, in the hippocampus it is not mainly glycine-receptor subtype 1alpha, but rather alpha 2 and alpha 3 that are expressed in hippocampal tissue (13, 19), which may weaken the presumption that glycine-receptor autoimmunity is involved in cognitive impairment in our patients, as they presented glycine 1alpha-receptor autoantibodies. Glycine in the hippocampus can result in glycine-mediated inhibition of the gamma amino butyric acid-based inhibition that in turn regulates neuronal activity in the hippocampus, which fine-tunes memory [for review see (17)]. Thus, glycine-receptor autoantibody might influence GABAergic signaling in the hippocampus, thereby affecting our patients' memory performance. The other possibility is the linking of glycine-receptor antibodies instead of glycine to binding sites on N-methyl-D-aspartate receptors. Glycine binding to NMDAR might result in NMDAR internalization (20). Thus, the linking of glycine-receptor autoantibodies to glycine-binding sites on NMDAR might result in internalizing NMDAR, thus disrupting excitatory synaptic transmission within the hippocampus to cause memory impairment. However, as glycine-receptor antibodies are often associated with brain-stem and spinal disorders (5) it is tempting to postulate additional mechanisms that would impair memory formation and recall mechanisms at the brainstem level. We also know that glycine receptors are expressed in the brainstem nucleus locus coeruleus (21). As the locus coeruleus is involved in memory formation in humans (22) we hypothesize that glycine-receptor autoimmunity at the locus coeruleus level might alter memory formation already at the brainstem level before the information reaches the parahippocampus and hippocampus.

However, we find it curious that memory is so severely impaired while other cognitive domains remain relatively mildly affected, like cognitive flexibility, although 75% of patients present a tauopathy that should be accompanied by frontal manifestations of tau deposits also. Furthermore, it is conceivable that it is not the autoantibodies *per se* that are relevant to memory impairment in our patients, but rather the tau depositions in limbic areas such as the hippocampus that could also induce memory impairment. We cannot rule out that our case series' tauopathy may also be age-related. It might help in future studies to compare MRI volumetric data on different regions of interest at the disease onset with follow-up MRIs to better delineate a relationship between neurodegeneration and glycine-receptor autoimmunity. The phenotypic appearance might be related also to how glycine-receptor autoantibodies bind to specific target regions, as there is evidence that glycine-receptor 1 alpha antibodies bind in the spinal cord and brainstem of patients with a PERM phenotype, as Carvajal-González et al. (13). The memory impairment or visual dysfunction in patients with glycine-receptor antibodies might thus be related to how glycine-receptor antibodies bind to specific brain regions. The focal brain atrophy in the hippocampus in one patient might also be an early Alzheimer's disease correlate, although that is unlikely as his CSF Aß42 and CSF-based Aß42/Aß40 ratio were normal. Further studies with structural-functional analysis should be done to better clarify this issue. Note that our case series is limited by its low patient numbers, which make us unable to draw conclusions about the relevance of a potentially novel phenotype of glycine-receptor autoimmunity. Furthermore, no relationship between glycine-receptor autoimmunity, neurodegeneration, tauopathy, and cognitive impairment can be derived from this case series study due to its design.

## Conclusion

Our case series demonstrate the occurrence of glycine receptor autoimmunity in patients with minor and major cognitive dysfunction as the leading symptom. Global cognitive function and verbal memory recall are often severely affected in these patients, while their visuoconstructive capacity remains practically unaffected. These phenotypic characteristics may help us further differentiate and select patients in memory clinics for certain diagnostics. Therapeutic approaches such as immunotherapy are classified as individual trial therapy according to proposed guidelines (11) if criteria such as axonal degeneration or brain atrophy or CSF inflammation are additionally present. None of our patients presented an obvious CNS inflammatory syndrome and were thus unlikely to be suffering from an acute inflammation. In addition, we detected no CSF glycine-receptor autoantibodies, nor any hint of intrathecal IgG synthesis. These facts weaken the hypothesis of causal involvement of glycine receptor-mediated autoimmunity in impaired memory recall. However, a chronic mild encephalitis might have contributed to the cognitive decline that is accompanied by a later axonal degeneration. More large cohort studies are needed to characterize the phenotype associated with glycine receptors and search for a relationship between glycine receptor antibodies and their phenotypic spectrum. Furthermore, the prevalence of glycine receptor-associated cognitive decline remains unknown, and the phenomenon is not understood. More large scale studies will help us to elucidate the frequency and significance of glycine-receptor associated cognitive impairment in memory clinic patients.

## Data Availability Statement

The data is available on demand from the corresponding author.

## Ethics Statement

The studies involving human participants were reviewed and approved by Ethics Committee University Medical Center Göttingen, Von-Siebold-Str. 3, 37075 Göttingen, Germany. Written informed consent for participation was not required for this study in accordance with the national legislation and the institutional requirements.

## Author Contributions

NH wrote and conceptualized the manuscript. CB, DF, JW, and WS revised the manuscript for important intellectual content. All authors contributed to the article and approved the submitted version.

## Conflict of Interest

The authors declare that the research was conducted in the absence of any commercial or financial relationships that could be construed as a potential conflict of interest.

## Publisher's Note

All claims expressed in this article are solely those of the authors and do not necessarily represent those of their affiliated organizations, or those of the publisher, the editors and the reviewers. Any product that may be evaluated in this article, or claim that may be made by its manufacturer, is not guaranteed or endorsed by the publisher.
